# Utilisation of mental health transfer checklist proforma from acute physical health hospitals (Liverpool University Hospitals NHS Foundation Trust) to mental health hospitals (Mersey Care NHS Foundation Trust)

**DOI:** 10.1192/bjo.2021.812

**Published:** 2021-06-18

**Authors:** Dalal Al-Bazz, Fareeba Anwar, Qaiser Javed

**Affiliations:** 1 Aintree Mental Health Liaison Team; 2 Royal Liverpool Mental Health Liaison Team; 3 Consultant Liaison Psychiatrist, Aintree University Hospital, Mental Health Liaison Team, Mersey Care NHS Foundation Trust

## Abstract

**Aims:**

Testing the compliance and completion rate of a transfer checklist (proforma) created in accordance with local hospital policies.

**Background:**

The proforma was developed following serious incidents where medically unstable patients were inappropriately discharged to mental health hospitals, requiring readmission to acute medical hospitals. Frequently these events reported an inadequate handover from medical to mental health teams and patients were often prematurely deemed medically fit with evidence to the contrary.

Although parity of esteem between mental and physical health has been a high profile political issue in the UK since 2011, evidence indicates that parity is far from being achieved. This first ever checklist was designed to improve safety of patient transfer from acute physical health hospitals to mental health hospitals by ensuring patients are medically fit and better communication between the two trusts.

**Method:**

Data were collected retrospectively over a six-month period between August 2018 and January 2019 and retrieved from patient notes available at relevant trusts. Electronic notes were obtained from medical wards, accident and emergency and Mersey Care electronic systems. Notes were specifically scrutinised for presence of the proforma, quality of completion and, number and reasons for readmission from mental health hospitals to acute physical health hospitals following their medical optimization. Readmissions were considered as admissions to physical health hospitals up to one month following discharge with evidence of ongoing concerns.

**Result:**

6597 referrals were made to liaison services from Liverpool University Hospitals, of which 5–6 % were admitted to inpatient mental health units. 31% of admissions from Liverpool University Hospitals were readmitted to a physical health hospital within one month of discharge indicating inappropriate and unsafe discharges. Of all those readmitted, 10% had ongoing acute medical concerns prior to admission to a mental health hospital. The proforma was filled in 13% of admissions from Liverpool University Hospitals. None of the forms were fully complete.

**Conclusion:**

10% of patient admissions to mental health hospitals were identified as inappropriate due to ongoing acute medical concerns. The proforma served as structured guidance and evidence of medical fitness at time of transfer. However poor compliance was observed, which could be secondary to lack of awareness of the proforma and inadequate dissemination of the policy. Findings were shared and discussed with the appropriate teams both in acute physical health and mental health hospitals and steps will be taken to raise awareness of the proforma before completing a second audit.

